# MDS-Related Anemia Is Associated with Impaired Quality of Life but Improvement Is Not Always Achieved by Increased Hemoglobin Level

**DOI:** 10.3390/jcm12185865

**Published:** 2023-09-09

**Authors:** Yael Haring, Noa Goldschmidt, Shaimaa Taha, Galia Stemer, Kalman Filanovsky, Ilana Hellman, Doaa Okasha, Baher Krayem, Itai Levi, Hanna Rosenbaum, Maya Koren-Michowitz, Shai Yagna, Anatoly Nemets, Sharon Gino-Moor, Revital Saban, Joseph Cohen, Erez Halperin, Ofir Wolach, Najib Dally, Drorit Merkel, Howard S. Oster, Moshe Mittelman

**Affiliations:** 1 Department of Hematology, Tel Aviv Sourasky Medical Center, Tel Aviv 6423906, Israel; yaelharing@gmail.com (Y.H.); noagold@tlvmc.gov.il (N.G.); 2 MDS Center, Tel Aviv Sourasky Medical Center, Tel Aviv 6423906, Israel; 3 Faculty of Medicine, Tel Aviv University, Tel Aviv 6997801, Israel; taha.l.shaimaa@gmail.com (S.T.); drorit.merkel@sheba.health.gov.il (D.M.); 4 Department of Internal Medicine, Tel Aviv Sourasky Medical Center, Tel Aviv 6423906, Israel; 5 Galillee Medical Center, Bar-Ilan University, Nahariya 5290002, Israel; galias@gmc.gov.il; 6 Kaplan Medical Center, Rehovot 7610001, Israel; kalmanph@clalit.org.il; 7 Meir Medical Center, Kfar Saba 4428164, Israel; hellmani@clalit.org.il; 8 Haemek Medical Center, Afula 1834111, Israel; doaaaka@clalit.org.il; 9 Rambam Health Care Campus, Haifa 3109601, Israel; b_krayem@rambam.health.gov.il; 10 Soroka Medical Center, Be’er Sheva 84101, Israel; etail@clalit.org.il; 11 Clalit Central Clinic, Nazareth 1641100, Israel; hanaro5@clalit.org.il; 12 Shamir Medical Center, Zerrifin 39040, Israel; korenm@asaf.health.gov.il; 13 Baruch Pade-Poriya Medical Center, Tiberias 1528001, Israel; 14 Barzilai Medical Center, Ashkelon 78306, Israel; anatolyn@barzi.health.gov.il; 15 Bney-Zion Medical Center, Haifa 3103301, Israel; sharon.ginomoor@b-zion.org.il; 16 Hadassah Medical Center, Jerusalem 9112001, Israel; sabanre@hadassah.org.il; 17 Laniado Medical Center, Netanya 4290200, Israel; yocohen@laniado.org.il; 18 Davidoff Cancer Center, Rabin Medical Center, Petah Tikva 4941492, Israel; erezha11@clalit.org.il (E.H.); ofirw@clalit.org.il (O.W.); 19 Ziv Medical Center, Bar-Ilan University, Zefad 5290002, Israel; nagib.d@ziv.health.gov.il; 20 MDS Center, Sheba Medical Center, Ramat Gan 5262000, Israel

**Keywords:** myelodysplastic syndrome, myelodysplastic neoplasms, anemia, quality of life, transfusion

## Abstract

Quality of life is impaired in MDS, but the role of hemoglobin level is unclear. To study the Hb–QoL correlation at diagnosis and 1 year later, patients filled out the EQ-5D questionnaire, assessing their mobility, self care, daily activities, pain/discomfort, and anxiety/depression, using scores of 0 (normal), 1 (mild/moderate), or 2 (poor). They also evaluated their health using a visual analogue scale, scoring from 0 (poor) to 100 (excellent). The anemia subgroups were: none/normal (Hb ≥ 12.5 g/dL), mild (10 ≤ Hb < 12.5), moderate (9 ≤ Hb < 10), severe (8 ≤ Hb < 9), or very severe (Hb < 8). LR-MDS patients (n = 127) and inpatient controls (n = 141) participated. The anemic patients had a poor QoL and the MDS patients had a lower QoL with a lower Hb. The controls had no QoL difference among the various anemia subgroups. In addition, the MDS QoL sharply decreased with an Hb of < 9. The MDS patients showed a wide QoL variability, i.e., different QoL scores in the same Hb subgroup, suggesting that other factors affect QoL (e.g., age and comorbidities). After 1 year (n = 61), the QoL was still poor for most MDS patients (including 27 patients with an increased Hb). In summary: (1) a poor QoL in MDS-anemia is non-linear, suggesting other influencing factors on QoL. (2) The sharp QoL drop with Hb < 9 g/dL challenges the transfusion Hb threshold. (3) The QoL in anemic MDS patients might differ from that in non-MDS patients. (4) Raising Hb, while recommended, does not guarantee an improved QoL.

## 1. Introduction

Myelodysplastic syndromes or neoplasms (MDS) are a heterogenous group of bone marrow (BM) clonal hematopoietic stem cell disorders [1,2,3,4]. MDS is characterized by impaired proliferation and differentiation, as well as BM failure. Cytopenias, especially anemia (90% of patients), are common and patients have a high propensity for leukemic transformation [3,4,5].

Health-related quality of life (HR-QoL), or QoL, is a broad term referring to objective and subjective aspects related to self human life as regarded by an individual [6,7].

Anemia is expected to be associated with an impaired QoL [8,9,10]. We, in the European MDS group [11,12], as well as others, have reported an impaired QoL in MDS patients compared to non-MDS controls.

The association between MDS-related anemia and impaired QoL does not necessarily mean causal relations, nor has the correlation between QoL and hemoglobin (Hb) level been fully elucidated. Is more severe anemia associated with a poorer QoL? Is this linear? Is Hb the only factor affecting QoL? Finally, the potential improvement in QoL, if and when treatment results in an increased Hb level, remains open. This was the focus of the current study.

The aims of the current study were: (1) to study the QoL at various degrees of anemia in MDS patients with lower-risk (LR) disease. (2) Compare this QoL with that of non-MDS controls. (3) Compare the QoL at MDS diagnosis to the QoL 1 year later.

## 2. Methods

### 2.1. Study Design & Patients

This is a retrospective study analyzing data collected as a part of the Israel National MDS registry, a part of the European MDS Registry [13]. In this real-world, non-interventional project, epidemiological, clinical, and lab data were collected and analyzed at presentation and every 6 months along the disease course. This LR-MDS patient population was categorized based on their Hb level: patients with no anemia/or normal Hb (Hb ≥ 12.5 g/dL), mild (10 ≤ Hb < 12.5), moderate (9 ≤ Hb < 10), severe (8 ≤ Hb < 9), and very severe anemia (Hb < 8 g/dL).

### 2.2. QoL

The MDS patients were requested to fill out the EQ-5D QoL questionnaire [14]. The questionnaire addresses 5 parameters reflecting various life aspects, as regarded by the patient: (1) mobility, (2) self care, (3) daily activities, (4) pain and/or discomfort, and (5) anxiety/depression. The patients were expected to subjectively score each parameter, while 0 meant excellent (normal), 1 reflected a mild/moderate decrease, and 2 indicated a poor status. The patients were then asked to report their general health status and provide a score, using a visual analogue scale (VAS) ranging from 0 (poor) to 100 (excellent QoL). To assess the QoL dynamics along the disease course, the patients filled out this survey every 6 months.

As controls, we used the VAS, as scored by consecutive patients, aged > 60 years, admitted to the Department of Medicine A, Tel Aviv Sourasky Medical Center (TASMC), from 1 to 15 March 2018. We were interested in their general quality of life in the period prior to admission.

### 2.3. Analysis

We compared among the 5 subgroups of anemic MDS patients with reference to QoL, as expressed by the mean EQ-5D and VAS scores. We then compared the QoL data (VAS) to those of the controls. Finally, we analyzed the QoL 1 year from MDS diagnosis, in an attempt to correlate between QoL and Hb.

### 2.4. Statistics

Two independent *t*-tests were performed to analyze the data. A *p* value of <0.05 was defined as statistically significant. The results were summarized in curves. Each dot represents the data of an individual subject and the linear curve represents the trend.

The study was approved by the local institutional review board (IRB) and the patients signed informed consent forms.

## 3. Results

In total, 127 consecutive patients participating in the Israel National MDS Registry were recruited and filled out the QoL EQ-5D questionnaire,. They were classified as having normal Hb/no anemia (n = 19), mild (n = 41), moderate (n = 17), severe (n = 21), and very severe anemia (n = 29), respectively. Table 1 displays the patient characteristics. The female/male ratio of the whole patient study population was 35/65%, and the mean age was 74.9 (36–91 years). As mentioned, all the patients were diagnosed as having lower-risk MDS.

**Figure 1:**
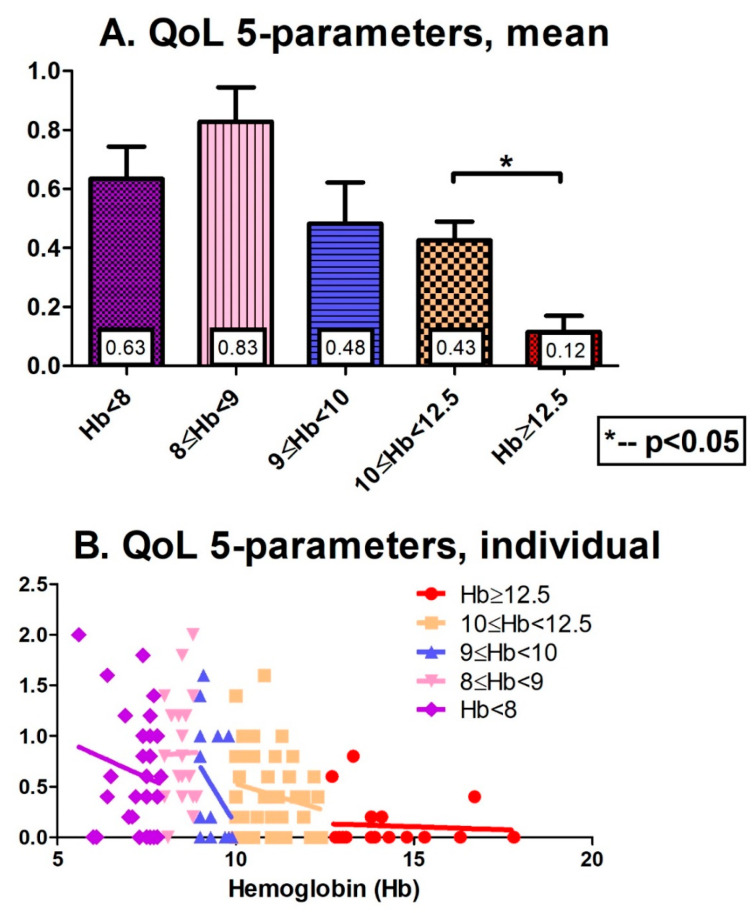
QoL scores (5 parameters) of the studied MDS patients. (**A**): The mean scores for the 5 subgroups: the mean EQ-5D scores were 0.12, 0.43, 0.48, 0.83, and 0.63 for normal, mild, moderate, severe, and very severe anemia, respectively. (**B**): The EQ-5D score for each individual patient, reflecting the large variability.

**Figure 2:**
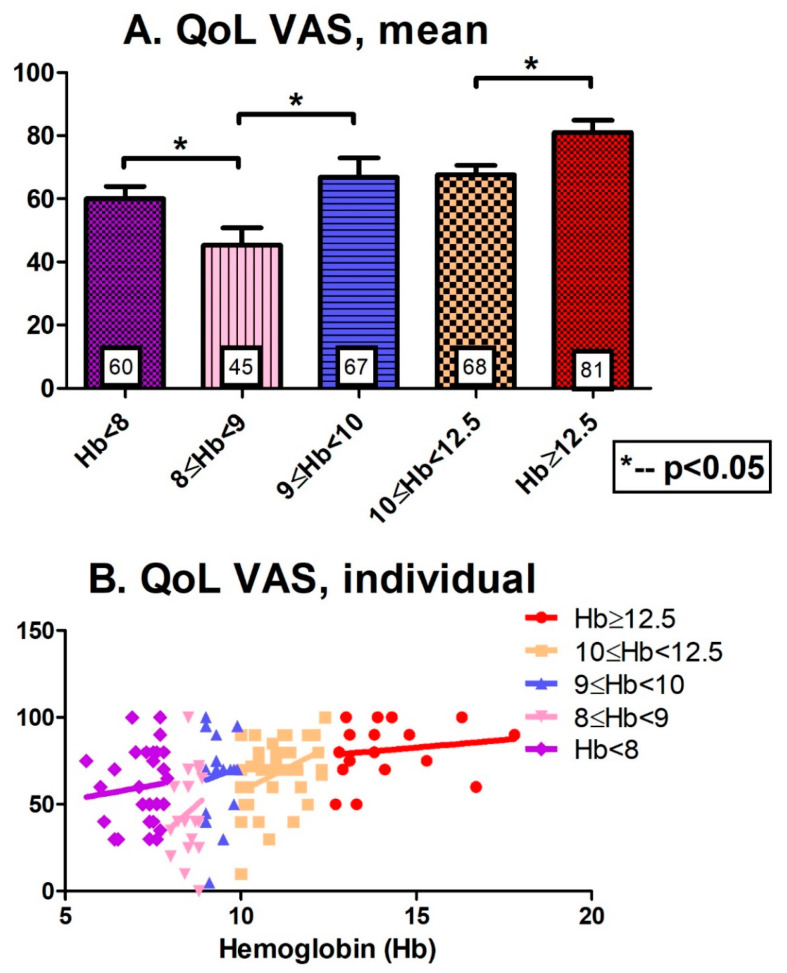
QoL scores (VAS) of the studied MDS patients. (**A**): The mean of the 5 various subgroups expressed as VAS score. (**B**): VAS score for each individual patient.

Figure 1 presents the QoL scores of the studied MDS patients. Figure 1A shows the mean scores for the five subgroups: The mean EQ-5D scores were 0.12, 0.43, 0.48, 0.83, and 0.63 for normal, mild, moderate, severe, and very severe anemia, respectively. Figure 1B displays the EQ-5D scores for each individual patient, reflecting the large variability. Figure 2A (mean of the various subgroups) and Figure 2B (individual score) display similar results for the five subgroups of MDS patients, expressed as the VAS score. In general, Hb decline was associated with impaired QoL, expressed by higher EQ-5D and lower VAS scores. It is noteworthy that the MDS patients experienced a wide QoL variability, i.e., patients belonging to the same anemia subgroup reported different QoLs (Figure 1B and Figure 2B). It is also worth noting the sharp drop in QoL from moderate to severe anemia (below 9 g/dL, *p* = 0.06 for EQ-5D score; *p* = 0.01 for VAS).

**Figure 3:**
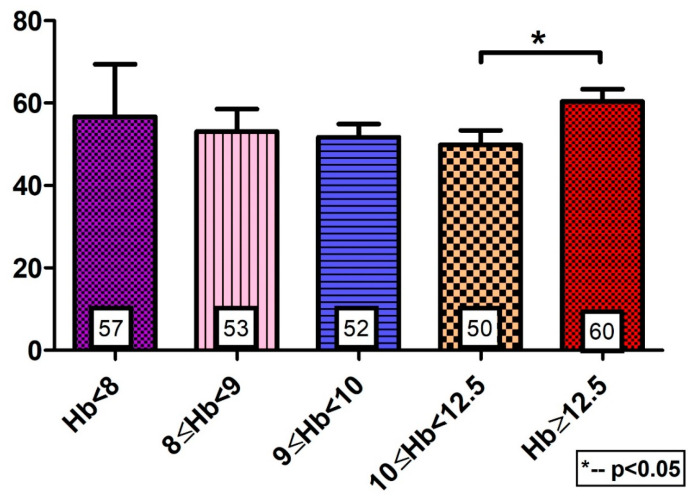
QoL VAS scores of non-MDS controls. While there is a difference in the VAS score between anemic and non-anemic patients, there is no significant difference among the anemic subgroups.

For comparison with non-MDS controls, we asked undiagnosed consecutive patients, aged > 60 years, admitted to the Department of Medicine, TASMC from 1 March 2018 through to 15 March 2018, to fill out the QoL-VAS questionnaires. The non-MDS controls (n = 141) also demonstrated lower VAS scores, i.e., impaired QoL, in anemic vs. non-anemic groups (VAS, Figure 3; *p* = 0.02). However, there was no difference among the anemic subgroups in the controls.

**Figure 4:**
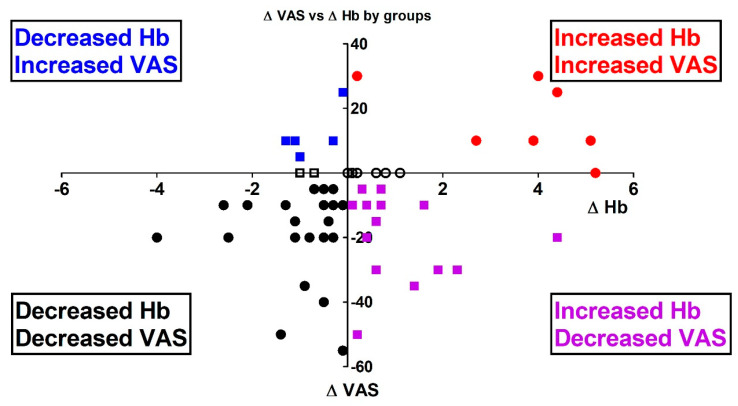
MDS patients, one year later. Individual Hb and QoL VAS scores 1 year after diagnosis for the 61 MDS patients for whom data were available. The right side represents an increased Hb level, and the left side a decreased Hb level. The upper part represents improved QoL (higher VAS score), while the lower part represents a declining QoL. Where the Hb decreased, 75% of them had a decreased VAS QoL score (left-lower quadrant, black dots); where the Hb increased, only 26% improved their QoL (right-upper quadrant, red dots). Please see text for details.

Follow-up QoL data, 1 year following diagnosis, were available for 61 MDS patients (Figure 4). The Hb results 1 year later: out of these patients, in 27 (44%), the Hb level rose, most probably due to treatment (red and purple, right side). In 32 (52%) patients, the Hb level declined (blue and black, left side), and 2 (3%) patients demonstrated no change in their Hb level.

QoL 1 year later in the MDS patients (Figure 4): of the 32 MDS patients, in those where the Hb level decreased after 1 year (left side), the QoL mean VAS score dropped by −11.88 [95% CI: −17.96, −5.79]. In 24 patients (75%) who suffered from a decreased Hb level, as expected, the VAS also decreased (Figure 3, left-lower quadrant, black dots). Interestingly, five patients (16%) still had an improved QoL or stayed the same (n = 3, 9%), despite the lower Hb level 1 year from diagnosis.

Of the 27 patients in whom the Hb level increased (right part, red and purple), the average VAS was still reduced by −6.48 [95% CI: −14.08, 1.12]. Seven patients (26%) improved their QoL (RUQ) or stayed the same (n = 5, 19%). Most of these patients (n = 15, 56%), despite the Hb level rise, still reported a decreased QoL VAS score (right-lower quadrant, purple dots).

## 4. Discussion

Over the last couple of decades, we have realized that in both practice as well as in research, in addition to measurable objective parameters such as response rate and survival, we should include subjective parameters regarded by patients, i.e., patient-reported outcomes (PRO) [15,16,17]. A major component of this is health-related quality of life (HR-QoL or QoL), which is now addressed in more reports and introduced as an endpoint in trials [7,9,16,18].

QoL is a broad term referring to objective (health status and welfare) and subjective (physical activity, feelings, fatigue, and frailty) aspects related to self human life, as regarded by an individual [6,9,15,17,19]. Being mainly subjective, evaluating and measuring QoL is challenging. The acceptable tool is patient questionnaires. This semi-objective method is not perfect, but allows for research, comparison between patients, and tge evaluation of medications. Several questionnaires have been applied, including Functional-Assessment of Cancer Therapy—Anemia (FACT-An) [6], Functional Assessment of Chronic Illness Therapy (FACIT) [20], the RAND 36-Item Health Survey questionnaire [21], the European Organisation for Research and Treatment of Cancer (EORTC) Quality of Life Questionnaire Core 30 items (QLQ-C30) [9,22], EORTC Quality of Life Utility—Core 10 Dimensions (QLU-C10D) [23], and others. Abel et al. developed a specific MDS-related questionnaire, defined as the Quality of Life in Myelodysplasia Scale (QUALMS) [24]. The survey, addressing aspects such as emotional stress, economic benefits, and others, was successfully applied in North America [25] and more recently by us, testing 270 patients from the European Registry, suggesting that QUALMS might be more appropriate for future clinical trials on MDS [11].

In this study, we applied the EQ-5D (three levels), a generic measure of health status that provides a simple descriptive profile and single index value that can be used in the clinical and economic evaluation of healthcare and in population health surveys [14,26]. Currently, EQ-5D is being widely used in different countries by clinical researchers in a variety of clinical areas. It is also used by regulators, as well as by the pharmaceutical industry. The questionnaire has been translated into numerous languages, and patients are requested to score their health status by providing a score to each of five aspects: mobility, self-care, daily activities, pain/discomfort, and anxiety/depression. Herdman et al. proposed a more detailed version of the questionnaire, the EQ-5D-5L, in which five instead of three scores are given to each evaluated parameter [27]. However, whether the proposed method is superior remains to be tested [28,29].

Despite accepting the idea that the relief of symptoms and improving QoL should be major goals of treatment, at least in lower-risk MDS [4,5,13], little has been conducted in terms of studying the topic or defining it as a major endpoint in clinical trials [9,16,30,31].

A few studies, mainly retrospective, have tested the QoL in MDS patients. We, in the EUMDS group, applied the EQ-5D at initial diagnosis to 1690 consecutive IPSS-Low/Int-1 MDS patients from the European LeukemiaNet Registry [12,32]. Impairments were compared with age- and sex-matched EuroQol Group controls. A significant proportion of MDS patients, more than the controls, reported moderate/severe problems in the dimensions of pain/discomfort (49.5%), mobility (41.0%), anxiety/depression (37.9%), and usual activities (36.1%). Other investigators have reported similar data, specifying fatigue and transfusion-dependence as QoL-related symptoms in this patient population [10,17,33,34]. Impaired QoL imposes an economic burden as well [33].

Many factors might affect QoL. Anemia has been traditionally considered as a major one. Oliva et al. reported a close correlation between a lower Hb level, cardiovascular morbidity and mortality, and impaired QoL [8]. Wouters et al. initiated a real-world, prospective Dutch-population-based survey, the Lifelines project, and in studying the data of 138,670 subjects, they concluded that anemia in individuals older than 60 years was associated with a shorter overall survival and impaired QoL [21,35]. However, whether anemia is the only or even the major factor influencing QoL remains unclear.

Anemia is the hallmark of MDS, found in more than 90% of patients [3,4,5]. It is also considered to be a prognostic marker [36]. Less has been reported on the role or relation between anemia severity and QoL in these patients. Lower Hb levels and red blood cell transfusion dependence are associated with a worse QoL [7,12,17,37]. However, despite the major role anemia has in QoL, there is a paucity of evidence on the degree of anemia or Hb level in relation to QoL.

In the current work, we focused on the relation between Hb level or anemia subgroup and QoL in lower-risk patients. The EQ-5D was quite reliable and confirmed the general tendency for an impaired QoL in these patients as the Hb level declined. Thus, we confirmed previous publications [7,10,12,15].

Of special interest, and in contrast with non-MDS controls, was the abrupt drop of QoL in anemic MDS patients with a Hb level below 9 g/dL. This finding, if confirmed in larger numbers and additional trials, is clinically significant. Today, the general therapeutic approach is quite restrictive, i.e., transfusing MDS (and other) patients only when their Hb level declines below 8 g/dL [4,13,38]. Our finding of an abrupt impairment in QoL below an Hb level of 9 g/dL might call for a second thought. These findings may lead to a paradigm shift towards a more liberal therapeutic approach, i.e., considering RBC transfusion if and when the Hb level deteriorates below the 9 g/dL threshold. This is supported by several recent publications showing that a more liberal approach, with maintaining the Hb level in the range of 11–12.5 g/dL, rather than > 8 g/dL, in MDS patients, is associated with an improved QoL [39]. A recent Canadian trial also found that a liberal transfusion policy was associated with an improved QoL [40]. Interestingly, a Delphi questionnaire obtained only a 75% expert concordance regarding the pre-transfusion Hb threshold (7.5 g/dL) but a 100% agreement about the safety of RBC transfusion if Hb > 7.5 g/dL [38]. A multi-national survey of patients with MDS requiring RBC transfusions demonstrated substantial variation in the patients’ experiences and preferences that differed also by country, supporting the need for further comparative clinical trials of transfusion practice interventions [41]. Thus, the recommended pre-transfusion Hb threshold remains an open question. However, transfusing at a lower Hb threshold does not guarantee an improved QoL (see below).

The large QoL variability reported by our patients, i.e., patients belonging to the same Hb subgroup reporting different levels of QoL, deserves further attention. It suggests that Hb might be important, but is not the only factor affecting QoL. As other publications have suggested, additional variables might affect QoL, such as an older age, female sex, comorbidities, a high body mass index, and physical function and performance status, as well as RBC transfusion need, leukocytosis, neutrophilia, lymphocytosis, monocytosis, thrombocytosis, a high neutrophil to lymphocyte ratio, and high serum ferritin level [12,29,34,42,43,44,45]. This will have to be taken into consideration in the future when designing therapies intended to improve QoL.

Of note, QoL following 1 year from diagnosis showed a general tendency of a continuing decline in QoL. Moreover, even patients who experienced an Hb rise reported an impaired QoL, further supporting the idea that the Hb level is only one out of several influencing factors. This finding, i.e., Hb elevation is not automatically associated with improved QoL, is supported by Abel et al. [46]. Oliva et al. concluded from the MEDALIST trial, in which luspatercept was administered to ESA-resistant patients with MDS and sideroblastic anemia, that improved QoL could be related not to a direct Hb rise but, to decreased transfusion needs [16]. Thus, raising the Hb level may improve QoL, but it may not, owing to other factors.

This research has several limitations. First, the inherent nature of patient questionnaires is semi-objective at best, and there are several confounders that should be taken into consideration. Some investigators believe that the EQ-5D is inferior to other questionnaires for MDS, such as the QLU-C10D [22,23] or QUALMS [11,15,24]. In addition, the numbers of the analyzed patients were relatively small.

In addition, the control group was not optimal. Their QoL could have been affected by other factors, including hospitalization. While our goal was to assess patients’ QoL during the period prior to admission, we suspect that their answers were affected by the hospitalization itself. Finally, other factors such as age, comorbidities, medications, and (other) MDS treatments that might influence QoL were not considered.

Nevertheless, this study delivers several messages. We confirmed that the QoL is indeed impaired in MDS patients, especially as the Hb declines. The sharp drop in QoL level with an Hb level below 9 g/dL, if confirmed, might lead to a therapeutic paradigm shift and a more liberal Hb threshold for RBC transfusion. The non-linear curve(s) and large variability in the QoL scores in the patients belonging to the same Hb subgroup suggest that other factors, in addition to Hb, might contribute to impaired QoL and pave the way for future research in the field. Finally, the 1 year follow-up QoL scores, despite the small numbers, further support the multifactorial nature of QoL in these patients. We believe that such reports, as well as others [7,17,42,45], will further support putting the topic of QoL on the general medical research agenda in clinical trials and prognostication and eventually will lead to the development of better and more effective therapeutic approaches.

## Figures and Tables

**Table 1:** Patient characteristics.

	All Patients	No Anemia/Normal	Mild Anemia	Moderate Anemia	Severe Anemia	Very Severe Anemia
**n**	**127**	19	41	17	21	29
**F/M**	**44/81** (35%/65%)	**7/12** (37%/63%)	**17/24** (41%/59%)	**7/10** (41%/59%)	**3/18** (15%/85%)	**12/17** (41%/59%)
**Age mean** **(range)**	**74.9** (36–91)	**67.6** (36–87)	**77.3** (62–89)	**75.1** (57–88)	**75.1** (40–91)	**75.8** (57–90)
**Hb, Mean** (g/dL)	10.0	14.1	11.0	9.4	8.5	7.2
**Mean MCV** (Fl)	95.2	90.9	95.6	98.0	95.0	96.0

Definition of anemia subgroups: no anemia/normal Hb ≥ 12.5 g/dL; mild (10 ≤ Hb < 12.5); moderate (9 ≤ Hb < 10); severe (8 ≤ Hb < 9); and very severe anemia (Hb < 8 g/dL).

## Data Availability

The data will be available upon request of the corresponding authors.
